# Radiomics: the facts and the challenges of image analysis

**DOI:** 10.1186/s41747-018-0068-z

**Published:** 2018-11-14

**Authors:** Stefania Rizzo, Francesca Botta, Sara Raimondi, Daniela Origgi, Cristiana Fanciullo, Alessio Giuseppe Morganti, Massimo Bellomi

**Affiliations:** 1 Department of Radiology, IEO, European Institute of Oncology, IRCCS, Milan, IT Italy; 2 Medical Physics, European Institute of Oncology, Milan, Italy; 3 Division of Epidemiology and Biostatistics, European Institute of Oncology, Milan, Italy; 4 Università degli Studi di Milano, Postgraduate School in Radiodiagnostics, Milan, Italy; 5 Radiation Oncology Center, School of Medicine, Department of Experimental, Diagnostic and Specialty Medicine – DIMES, University of Bologna, Bologna, Italy; 6 Department of Oncology and Hemato-Oncology, Università degli Studi di Milano, Milan, Italy

**Keywords:** Clinical decision-making, Biomarkers, Image processing (computer-assisted), Radiomics, Texture analysis

## Abstract

*Radiomics* is an emerging translational field of research aiming to extract mineable high-dimensional data from clinical images. The radiomic process can be divided into distinct steps with definable inputs and outputs, such as image acquisition and reconstruction, image segmentation, features extraction and qualification, analysis, and model building. Each step needs careful evaluation for the construction of robust and reliable models to be transferred into clinical practice for the purposes of prognosis, non-invasive disease tracking, and evaluation of disease response to treatment. After the definition of *texture* parameters (shape features; first-, second-, and higher-order features), we briefly discuss the origin of the term *radiomics* and the methods for selecting the parameters useful for a radiomic approach, including cluster analysis, principal component analysis, random forest, neural network, linear/logistic regression, and other. Reproducibility and clinical value of parameters should be firstly tested with *internal cross-validation* and then validated on *independent external cohorts*. This article summarises the major issues regarding this multi-step process, focussing in particular on challenges of the extraction of radiomic features from data sets provided by computed tomography, positron emission tomography, and magnetic resonance imaging.

## Key points

- Radiomics is a complex multi-step process aiding clinical decision-making and outcome prediction
- Manual, automatic, and semi-automatic segmentation is challenging because of reproducibility issues
- Quantitative features are mathematically extracted by software, with different complexity levels
- Reproducibility and clinical value of radiomic features should be firstly tested with internal cross-validation and then validated on independent external cohorts

## Background

In the new era of *precision medicine*, *radiomics* is an emerging translational field of research aiming to find associations between qualitative and quantitative information extracted from clinical images and clinical data, with or without associated gene expression to support evidence-based clinical decision-making [1]. The concept underlying the process is that both morphological and functional clinical images contain qualitative and quantitative information, which may reflect the underlying pathophysiology of a tissue. Radiomics’ analyses can be performed in tumour regions, metastatic lesions, as well as in normal tissues [2].

The radiomics quantitative features can be calculated by dedicated software, which accepts the medical images as an input. Despite many tools developed for this specific task being user-friendly in terms of use, and well performing in terms of calculation time, it is still challenging to carefully check the quality of the input data and to select the optimal parameters to guarantee a reliable and robust output.

The quality of features extracted, their association with clinical data, and also the model derived from them, can be affected by the type of image acquisition, postprocessing, and segmentation.

This article summarises the major issues regarding this multi-step process, focussing in particular on the challenges that the extraction and radiomics’ use of imaging features from computed tomography (CT), positron emission tomography (PET), and magnetic resonance imaging (MRI) generates.

## Definition and extraction of image features

Different kind of features can be derived from clinical images. Qualitative semantic features are commonly used in the radiology lexicon to describe lesions [3]. Quantitative features are descriptors extracted from the images by software implementing mathematical algorithms [4]. They exhibit different levels of complexity and express properties firstly of the lesion shape and the voxel intensity histogram, secondarily of the spatial arrangement of the intensity values at voxel level (*texture*). They can be extracted either directly from the images or after applying different filters or transforms (e.g., wavelet transform).

Quantitative features are usually categorised into the following subgroups:

*Shape features* describe the shape of the traced region of interest (ROI) and its geometric properties such as volume, maximum diameter along different orthogonal directions, maximum surface, tumour compactness, and sphericity. For example, the surface-to-volume ratio of a spiculated tumour will show higher values than that of a round tumour of similar volume.

*First-order statistics features* describe the distribution of individual voxel values without concern for spatial relationships. These are histogram-based properties reporting the mean, median, maximum, minimum values of the voxel intensities on the image, as well as their skewness (asymmetry), kurtosis (flatness), uniformity, and randomness (entropy).

*Second-order statistics features* include the so-called *textural features* [5, 6], which are obtained calculating the statistical inter-relationships between neighbouring voxels [7]. They provide a measure of the spatial arrangement of the voxel intensities, and hence of intra-lesion heterogeneity. Such features can be derived from the grey-level co-occurrence matrix (GLCM), quantifying the incidence of voxels with same intensities at a predetermined distance along a fixed direction, or from the *Grey-level run-length matrix* (GLRLM), quantifying consecutive voxels with the same intensity along fixed directions [8].

*Higher-order statistics features* are obtained by statistical methods after applying filters or mathematical transforms to the images; for example, with the aim of identifying repetitive or non-repetitive patterns, suppressing noise, or highlighting details. These include fractal analysis, Minkowski functionals, wavelet transform, and Laplacian transforms of Gaussian-filtered images, which can extract areas with increasingly coarse texture patterns.

Considering that many parameters can be tuned by the user, hundreds of variables can be generated from a single image.

Most of the abovementioned features are neither original nor innovative descriptors. Indeed, the definition and use of textural features to quantify image properties, as well as the use of filters and mathematical transforms to process signals, date back a few decades [6]. Therefore, the main innovation of radiomics relies on the –omics suffix, originally created for molecular biology disciplines. This refers to the simultaneous use of a large amount of parameters extracted from a single lesion, which are mathematically processed with advanced statistical methods under the hypothesis that an appropriate combination of them, along with clinical data, can express significant tissue properties, useful for diagnosis, prognosis, or treatment in an individual patient (personalisation). Additionally, radiomics takes advantage of the full use of large data-analysis experience developed by other -omics disciplines, as well as by big-data analytics.

Some difficulties arise when the user has to choose which and how many parameters to extract from the images. Each tool calculates a different number of features, belonging to different categories, and the initial choice may appear somehow arbitrary. Nonetheless, methods for data analysis strictly depend on the number of input variables, possibly affecting the final result. One possible approach is to start from all the features provided by the calculation tool, and to perform a preliminary analysis to select the most repeatable and reproducible parameters; to subsequently reduce them by correlation and redundancy analysis [9]. Another approach is to make an *a priori* selection of the features, based on their mathematical definition, focussing on the parameters easily interpretable in terms of visual appearance, or directly connectable to some biological properties of the tissue.

Alternatively, *machine-learning* techniques, underlying the idea that computers may learn from past examples and detect hard-to-discern patterns from large and complex data sets, are emerging as useful tools that may lead to the selection of appropriate features [10–12].

## Analysis and model building

Many of the extracted features are redundant. Therefore, initial efforts should focus on identifying appropriate endpoints with a potential clinical application, to select information useful for a specific purpose. Radiomics’ analysis usually includes two main steps:

1. Dimensionality reduction and feature selection, usually obtained via unsupervised approaches; and
2. Association analysis with one or more specific outcome(s) via supervised approaches.

**Fig. 1:**
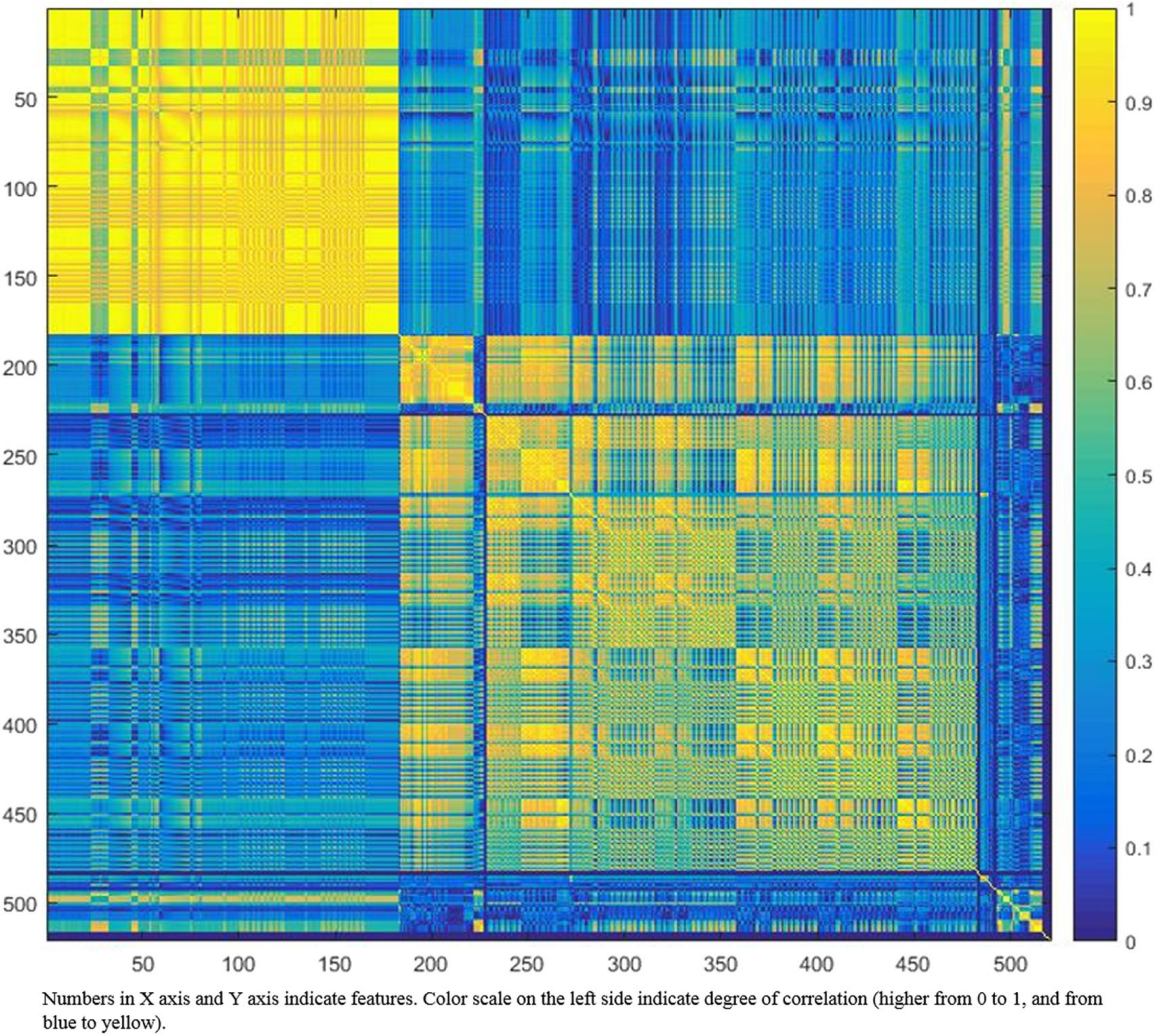
Graphic representation of radiomic-feature clustering. This example graph displays the absolute value of the correlation coefficient (ranging from 0 to 1, on the right side, indicating increasing degree of correlation) between each pair of radiomic features (shown as numbers on the two axes). The heat map gives a good visual representation of the high correlation observed for most radiomic features that may be grouped in the same cluster to avoid redundancy. The yellow blocks along the diagonal graphically identify the clusters including highly correlated radiomic features. Blue blocks outside the diagonal visualise the low correlation observed between radiomic features belonging to different clusters. In the present example, two major clusters with different information may be identified, with very high redundancy for radiomic features in the first cluster (high homogeneity of the yellow blocks)

Different methods of dimensionality reduction/feature selection and model classification have been compared [13, 14]. The two most commonly used unsupervised approaches are *cluster analysis* [7, 14, 15] and *principal component analysis* (PCA) [13, 16]. Cluster analysis aims to create groups of similar features (clusters) with high intra-cluster redundancy and low intercluster correlation. This type of analysis is usually depicted by a cluster heat map [17], as shown in Fig. 1. A single feature may be selected from each cluster as representative and used in the following association analysis [14, 15]. PCA aims to create a smaller set of maximally uncorrelated variables from a large set of correlated variables, and to explain as much as possible of the total variation in the data set with the fewest possible principal components [18]. Graphically, the output of PCA consists of score plots, giving an indication for grouping in the data sets for similarity.

All selected features considered reproducible, informative, and non-redundant can then be used for association analysis. According to our experience, an important *caveat* for univariate analysis is multiple testing. The most common way to overcome the multiple testing problem is to use Bonferroni correction or the less conservative false discovery rate corrections [19].

Supervised multivariate analysis consists of building a mathematical model to predict an outcome or response variable. The different analysis approaches depend on the purpose of the study and the outcome category, ranging from statistical methods to data-mining/machine-learning approaches, such as random forests [14, 20], neural networks [21], linear regression [21], logistic regression [15], least absolute shrinkage and selection operator [22], and Cox proportional hazards regression [23]. Previous studies comparing different model-building approaches found that the random forest classification method had the highest prognostic performance [13, 14].

Unquestionably, the stability and reproducibility of the model must be assessed before applying a predictive model in a clinical setting. Indeed, it is well known that model fitting is optimal in the training set used to build the model, while validation in an external cohort provides more reliable fitting estimates [24]. The first step in model validation is *internal cross-validation*. However, the best way to assess the potential clinical value of a model is validation with prospectively collected independent cohorts, ideally within clinical trials. This introduces the issue of data sharing among different institutions, creating the need for shared databases to be used as validation sets. To help solve this issue, there are large, publicly available databases, such as The Cancer Genome Atlas (TCGA), including comprehensive multidimensional genomic data and clinical annotations of more than 30 types of cancer [25]. Likewise, the Cancer Imaging Archive is a publicly available resource hosting the imaging data of patients in the TCGA database. These images can be used as valuable sources for both hypothesis generating and validation purposes [26].

Notably, patient parameters may influence image features via a direct causal association or exert a confounding effect on statistical associations. For instance, smoking-related lung cancers differ from lung cancers in non-smokers [27].

Moreover, since models need validation to be preferably performed on external and independent groups of patients, the comparability of features extracted from images with different parameters and segmented with different techniques is challenging and may affect the final performance of the model itself.

## Impact of image acquisition and reconstruction

**Fig. 2:**
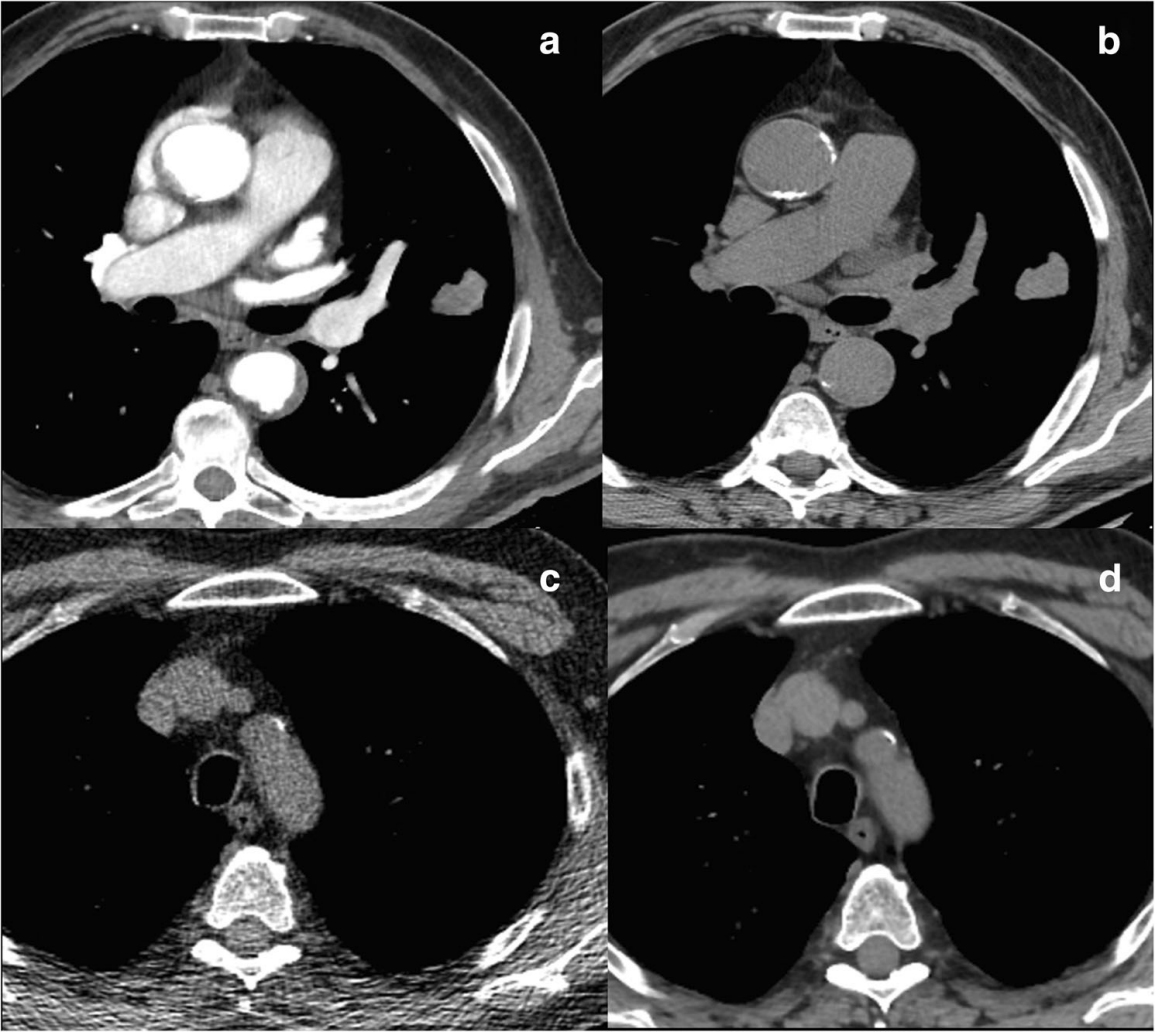
Axial computed tomography images showing differences in the same acquisition plane between a contrast-enhanced (**a**) and an unenhanced image (**b**), as well as for different radiation doses, lower in (**c**), and higher in (**d**)

Routine clinical imaging techniques show a wide variation in acquisition parameters, such as: image spatial resolution; administration of contrast agents; kVp and mAs (among others) for CT **(**Fig. 2**)**; type of sequence, echo time, repetition time, number of excitations and many other sequence parameters for MRI. Furthermore, different vendors offer different reconstruction algorithms, and reconstruction parameters are customised at each institution, with possible variations in individual patients. All these variables affect image noise and texture, and consequently the value of the radiomic features. As a result, features obtained from images acquired at a single institution using different acquisition protocols, or acquired at different institutions with different scanners in different patient populations, may be affected by different parameters, rather than reflecting different biological properties of tissues. Finally, some acquisition and reconstruction settings may yield to unstable features, thus showing different values when extracted from repeated measurements under identical conditions.

An approach to overcome this limitation may be to exclude from the beginning the features highly influenced by the acquisition and reconstruction parameters. This can be achieved by integrating information from the literature and from dedicated experimental measurements, taking into account the peculiarity of each imaging modality.

### CT

Standard CT phantoms, like those proposed by the American Association of Physicists in Medicine [28], allow the evaluation of imaging performance and the assessment of how far image quality depends on the adopted technique. Despite not being intended for this, they may provide useful information on the parameters potentially affecting image texture. For instance, a decrease in slice thickness reduces the photon statistics within a slice (unless mAs or kVp are increased accordingly), thereby increasing image noise. The axial field of view and reconstruction matrix size determine the pixel size and hence the spatial sampling in the axial plane, which has an impact on the description of heterogeneity. The reduction of pixel size increases image noise (when the other parameters are kept unchanged), but increases spatial resolution.

When considering spiral CT acquisition, pitch is a variable that influences image noise, making difficult the comparison between different scanners and vendors. Thus, non-spiral (axial) acquisitions are necessary for these comparisons. Likewise, clinical conditions, such as the presence of artifacts due to metallic prostheses, may affect image quality and impair quantitative analysis [29]. Furthermore, electronic density quantification expressed as Hounsfield Units may vary with the reconstruction algorithm [30] or scanner calibration.

Thus, to study in detail the effects of acquisition settings and reconstruction algorithms on radiomic features, more sophisticated phantoms are required. For example, the Credence Cartridge Radiomics phantom, including different cartridges, each of them exhibiting a different texture, was developed to test inter-scanner, intra-scanner, and multicentre variability [31], as well as the effect of different acquisition and reconstruction settings on feature robustness [4]. Another possibility is to develop customised phantoms [32] resembling the anatomic districts of interest, embedding inserts simulating tissues with different texture and size, and located at different positions, to test protocols under real clinical conditions.

Alternatively, many authors have investigated features of robustness and stability on clinical images by undertaking test-retest studies [33], or comparing the results obtained with different imaging settings and processing algorithms [34]. These studies conclude that there is still the need for dedicated investigations to select features with sufficient dynamic range among patients, with intra-patient reproducibility and low sensitivity to image acquisition and reconstruction protocols [15].

### PET

Texture analysis on PET images poses additional challenges. PET spatial resolution is in general worse than that of CT, because of low accuracy in describing the spatial distribution of VI, which radiomic features aim to quantify. This relies on different physical phenomena, different technologies used for radiation detection, and patient motion. Less accurate data may fail in generating significant association with biological and clinical endpoints, or may require an increased number of patients.

Of note, the VI, expressed in terms of standardised uptake value (SUV) can be scanner dependent. For example, modelling or not the detector response in the reconstruction algorithm leads to a lymph node SUV_mean_ difference of 28% [35]. Furthermore, for the same scanner model, SUV differences (hence radiomic-feature differences) may be due to acquisition at different times post injection, patient blood glucose level and presence of inflammation [36].

Previous studies provided data to select the most appropriate procedures and radiomic PET features [37–39]. For example, voxel size was shown to be the most important source of variability for a large number of features, whereas the *entropy* feature calculated from the GLCM was robust with respect to acquisition and reconstruction parameters, post-filtering level, iteration number, and matrix size [35].

For dedicated experimental measurements, phantoms routinely used for PET scanner quality control may be used. For instance, the NEMA Image Quality phantom has been used to assess the impact of noise on textural features when varying reconstruction settings [37, 40], whereas homogeneous phantoms have been used to test stability [41]. To our knowledge, commercial phantoms customised for testing radiomic-feature performance in the presence of inhomogeneous activity distributions are not yet available, but home-made solutions have been described [41].

Scanner calibration and protocol standardisation are necessary to allow for multicentre studies and model generalisability [9, 42]. Harmonisation methods are emerging to allow gathering and comparing data from different centres, although they are not yet largely applied in clinical studies [35].

### MRI

**Fig. 3:**
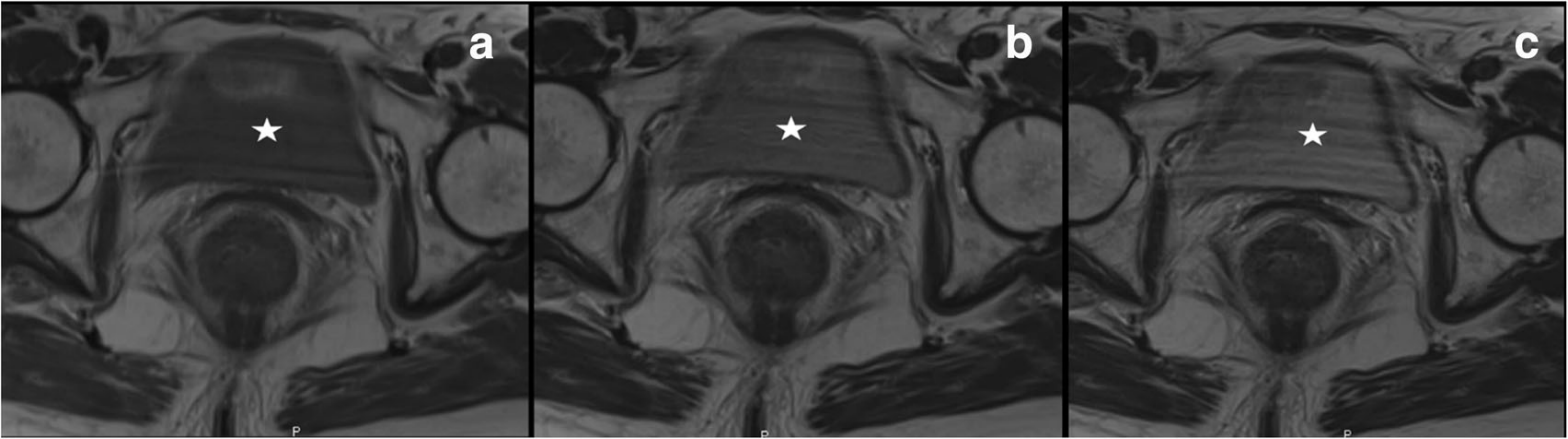
Axial T2-weighted images of the pelvis, acquired keeping unchanged all the parameters, with only exception of the echo time, which was 34 ms in (**a**), 90 ms in (**b**), and 134 ms in (**c**), showing that even one single parameter can change the signal intensity of tissues and fluids, as clearly depicted by the signal of the bladder (white star), with higher and higher signal intensity from **a** to **b** to **c**

The signal intensity in MRI arises from a complex interaction of intrinsic tissue properties, such as relaxation times as well as multiple parameters related to scanner properties, acquisition settings, and image processing. For a given T1- or T2-weighted sequence, voxel intensity does not have a fixed tissue-specific numeric value. Even when scanning the same patient in the same position with the same scanner using the same sequence in two or more sessions, signal intensity may change (Fig. 3), whereas tissue contrast remains unaltered [43].

Without a correction for this effect, a comparison of radiomic features among patients may lose significance as it depends on the numeric value of voxel intensity. One possibility is to focus texture analysis on radiomic features quantifying the relationship between voxel intensities, where numerical values do not depend on the individual voxel intensity; another is to make a compensation (normalisation) before performing quantitative image analysis [43].

Current studies investigating the impact of MRI acquisition parameters on radiomic-feature robustness address the complexity of the technique and the low availability of proper phantoms. The available data suggest that texture features are sensitive to variations of acquisition parameters: the higher the spatial resolution, the higher the sensitivity [44]. A trial assessing radiomic features obtained on different scanners at different institutions or with different parameters concluded that comparisons should be treated with care [45].

## Impact of image segmentation

**Fig. 4:**
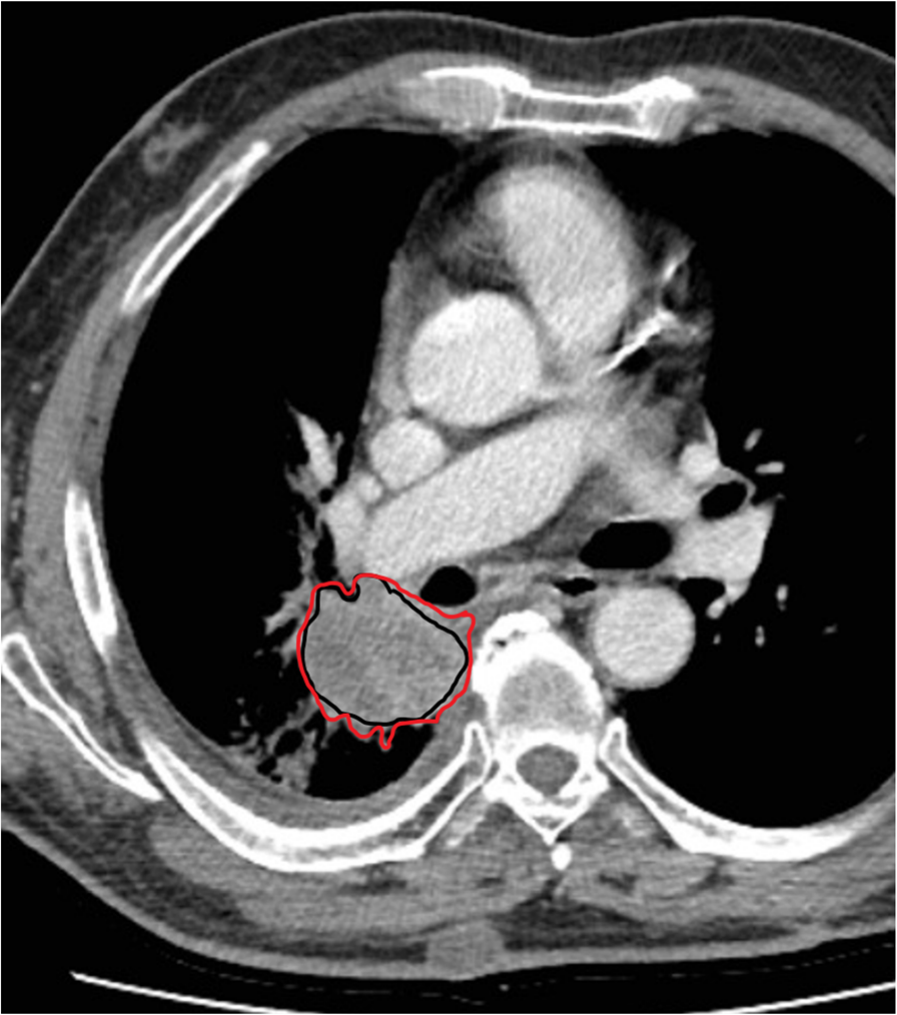
An example of manual segmentation of lung cancer on computed tomography images. Although manual segmentation is often considered ground truth, this image shows red and black regions of interest delineated by two different readers for the same tumour

Segmentation is a critical step of the radiomics process because data are extracted from the segmented volumes. This is challenging because many tumours show unclear borders. It is contentious because there is no consensus on the need to seek either the ground truth or reproducibility of segmentation [1]. Indeed, many authors consider manual segmentation by expert readers the ground truth despite high inter-reader variability. This method is also labour intensive (Fig. 4) and not always feasible for radiomics’ analysis, requiring very large data sets [46].

Automatic and semi-automatic segmentation methods have been developed across imaging modalities and different anatomical regions. Common requirements include maximum automaticity with minimum operator interaction, time efficiency, accuracy, and boundary reproducibility. Some segmentation algorithms rely on region-growing methods that require an operator to select a seed point within the volume of interest [47]. These methods work well for relatively homogeneous lesions, but show the need for intensive user correction for inhomogeneous lesions. For example, most stage I and stage II lung tumours present as homogenous, high-intensity lesions on a background of low-intensity lung parenchyma [48, 49] and, therefore, can be automatically segmented with high reproducibility and accuracy. However, for partially solid, ground-glass opacities, nodules attached to vessels and to the pleural surface, automatic segmentation is burdened by low reproducibility [50].

Other segmentation algorithms include *level-set methods* that represent a contour as the zero-level set of a higher dimensional function (level-set function), then the method formulates the motion of the contour as the evolution of the level-set function [51]. *Graph-cut methods* construct an image-based graph and accomplish a globally optimal solution of energy minimisation functions, but they are computationally expensive [52] and may lead to over-segmentation [53]. *Active contour (snake) algorithms* work like a stretched elastic band. The starting points are drawn around the lesion; then move through an iterative process to a point with the lowest energy function value. These algorithms may lead the snake to undesired locations because they depend on an optimal starting point and are sensitive to noise [54]. Semi-automatic segmentation algorithms do a graph search through local active contour analysis, while their cost function is minimised using dynamic programming. Nonetheless, the semi-automaticity still requires human interaction [55].

As shown, there is still no universal segmentation algorithm for all image applications, and new algorithms are under evaluation to overcome these limitations [56–58]. Indeed, some features may show stability and reproducibility using one segmentation method, but not another.

## Conclusions

To summarise, staying in the present while looking into the future, on the one hand, investigators should put efforts in careful selection of robust features for their own models; on the other hand, the scientific community should put efforts towards standardisation, keeping in mind that appropriate statistical approaches will minimise spurious relationships and lead to more accurate and reproducible results.

These will be unavoidable steps towards the construction of generalisable prognostic and predictive models that will effectively contribute to clinical decision-making and treatment management.

## Abbreviations

CT: Computed tomography
GLCM: Grey-level co-occurrence matrix
GLRLM: Grey-level run-length matrix
MRI: Magnetic resonance imaging
PCA: Principal component analysis
PET: Positron emission tomography
ROI: Region of interest
SUV: Standardised uptake value
TCGA: The Cancer Genome Atlas
